# Distribution of Beta-Lactamase Producing Gram-Negative Bacterial Isolates in Isabela River of Santo Domingo, Dominican Republic

**DOI:** 10.3389/fmicb.2020.519169

**Published:** 2021-01-13

**Authors:** Víctor V. Calderón, Roberto Bonnelly, Camila Del Rosario, Albert Duarte, Rafael Baraúna, Rommel T. Ramos, Omar P. Perdomo, Luis E. Rodriguez de Francisco, Edian F. Franco

**Affiliations:** ^1^Instituto Tecnológico de Santo Domingo (INTEC), Santo Domingo, Dominican Republic; ^2^Institute of Biological Sciences, Federal University of Pará-UFPA, Belem, Brazil; ^3^Instituto de Innovación en Biotecnología e Industria (IIBI), Santo Domingo, Dominican Republic

**Keywords:** resistance genes, multidrug resistant, beta-lactamase, Isabela river, dominican republic, multidrug-resistant genomes, resistomes

## Abstract

Bacteria carrying antibiotic resistance genes (ARGs) are naturally prevalent in lotic ecosystems such as rivers. Their ability to spread in anthropogenic waters could lead to the emergence of multidrug-resistant bacteria of clinical importance. For this study, three regions of the Isabela river, an important urban river in the city of Santo Domingo, were evaluated for the presence of ARGs. The Isabela river is surrounded by communities that do not have access to proper sewage systems; furthermore, water from this river is consumed daily for many activities, including recreation and sanitation. To assess the state of antibiotic resistance dissemination in the Isabela river, nine samples were collected from these three bluedistinct sites in June 2019 and isolates obtained from these sites were selected based on resistance to beta-lactams. Physico-chemical and microbiological parameters were in accordance with the Dominican legislation. Matrix-assisted laser desorption ionization-time of flight mass spectrometry analyses of ribosomal protein composition revealed a total of 8 different genera. Most common genera were as follows: *Acinetobacter* (44.6%) and *Escherichia* (18%). Twenty clinically important bacterial isolates were identified from urban regions of the river; these belonged to genera *Escherichia* (*n* = 9), *Acinetobacter* (*n* = 8), *Enterobacter* (*n* = 2), and *Klebsiella* (*n* = 1). Clinically important multi-resistant isolates were not obtained from rural areas. Fifteen isolates were selected for genome sequencing and analysis. Most isolates were resistant to at least three different families of antibiotics. Among beta-lactamase genes encountered, we found the presence of bla_TEM_, bla_OXA_, bla_SHV_, and bla_KPC_ through both deep sequencing and PCR amplification. Bacteria found from genus *Klebsiella* and *Enterobacter* demonstrated ample repertoire of antibiotic resistance genes, including resistance from a family of last resort antibiotics reserved for dire infections: carbapenems. Some of the alleles found were KPC-3, OXA-1, OXA-72, OXA-132, CTX-M-55, CTX-M-15, and TEM-1.

## 1. Introduction

Despite significant pharmacological advances in the development of effective treatments against multidrug-resistant bacteria, antibiotic resistance remains a very imminent global health threat. The catastrophic reach of multi-resistant infections is represented in approximately 3 trillion dollars invested in therapy for patients affected worldwide (Ventola, 2015; Naylor et al., 2018). In 2016 alone, almost half a million people underwent severe infections by multidrug-resistant tuberculosis as reported by The World Health Organization (World Health Organization, 2018). The Centers for Disease Control and Prevention (CDC) of the United States categorized the global antibiotic resistance crisis as “a serious, worrying, and urgent threat” for the health of the population (CDC and prevention, 2013).

Antibiotic resistance genes (ARGs) are increasingly common in aquatic environments, especially genes of Extended Spectrum β-Lactamases (ESBLs) that are responsible for multi-resistant infections (Chouchani et al., 2013). One of the most urgent concerns in the study of ARGs is the ubiquity of ESBLs in urban rivers and lakes (Tacão et al., 2012; Freitas et al., 2019; Reddy and Dubey, 2019). The interaction between anthropogenic effluents (such as the hospital, abattoir, industrial, or residential wastewaters) and environmental waters such as rivers and lakes promotes the formation of resistance genes reservoirs (Marti et al., 2014). This increases the probability of horizontal gene transfer between clinically important bacteria and environmental species (Zhou et al., 2018). The confluence of anthropogenic effluents in rivers changes the distribution of ARGs because of the interaction between environmental and human ARGs (Rowe et al., 2017).

ESBLs are a family of enzymes conferring resistance to β-lactam antibiotics, such as penicillins, cephalosporins, and monobactam aztreonam. Over 90% of ampicillin-resistant *Escherichia coli* is attributed to the bla_TEM_ gene family, which is one of the most prevalent beta-lactamase genes commonly found in Gram-negative bacteria (Livermore, 1995; Ghafourian et al., 2015). Microorganisms such as *Klebsiella* spp. and *Enterobacter* spp. are usually observed harboring these genes (Bush and Jacoby, 2010). Carbapenemases are enzymes that catalyze the hydrolysis of many β-lactams such as penicillins, cephalosporins, monobactams, and most notably, carbapenems. The emergence of carbapenemase-producing bacteria in clinical settings introduced a significant public health concern due to the reduced spectrum of antibiotics available to medical professionals during the treatment of resistant bacterial infections. Several families of class C and class D carbapenemases have been strongly associated with bacteria commonly found in highly impacted aquatic environments; genes for carbapenemases have also been well-documented to be transmitted from one organism to another through mechanisms of genetic exchange that take place in the environment (Queenan and Bush, 2007; Abrantes, 2017; Alves et al., 2020). The sharply increasing prevalence of ARGs in environmental and clinical settings represents a serious risk to human health.

Due to the generalized absence of wastewater treatment systems in developing countries, it is especially common to encounter untreated effluents flowing directly into large rivers and lakes (Reddy and Dubey, 2019). This issue is of critical importance considering rivers and lakes under these conditions are reservoirs of diverse and abundant ARGs (Marti et al., 2014; Grenni et al., 2018). These environmental conditions are evident in the Isabela river of Santo Domingo, where river banks surrounded by communities inadequately deposit their waste into the stream and lack access to proper sewage systems. It is also valuable to consider the intense use of this river's waters by an important fraction of the city as their predominant source of daily consumption.

Isabela river (18°30′34.254″N,69°54′29.5668″W) is one of the fundamental sources of water of the city of Santo Domingo. This river can be found inside the metropolitan area, which is densely populated with over 3.8 million residents as estimated by the Statistics National Office (ONE, 2016). Also, many densely populated areas flank this watershed and utilize its resources for their living. The socioeconomic importance of the Isabela river was pivotal for its study. This river is constantly in contact with untreated effluents of illegal settlements on the river banks (Chantada, 1991; Gutiérrez, 2014). This is a serious public health concern considering that aquatic environments have been described as primal channels for horizontal transfer of ARGs with the human microbiome (Yelin and Kishony, 2018). This circumstance is aggravated by Dominicana's history of antibiotic abuse and its lack of regulation.

Isabela river holds an active abattoir and a metal processing factory; these industries have been previously described as factors that alter the prevalence of ARGs in the environment (Araújo et al., 2010; Andrade et al., 2018). Based on these characteristics and conditions, this study aims to describe the diversity of resistance genes found in the Isabela river, specifically, ESBLs, a family of ARGs that target beta-lactam antibiotic, widely used by the Dominican population.

## 2. Materials and Methods

### 2.1. Sampling and Physico-Chemical Analyses

**Figure 1:**
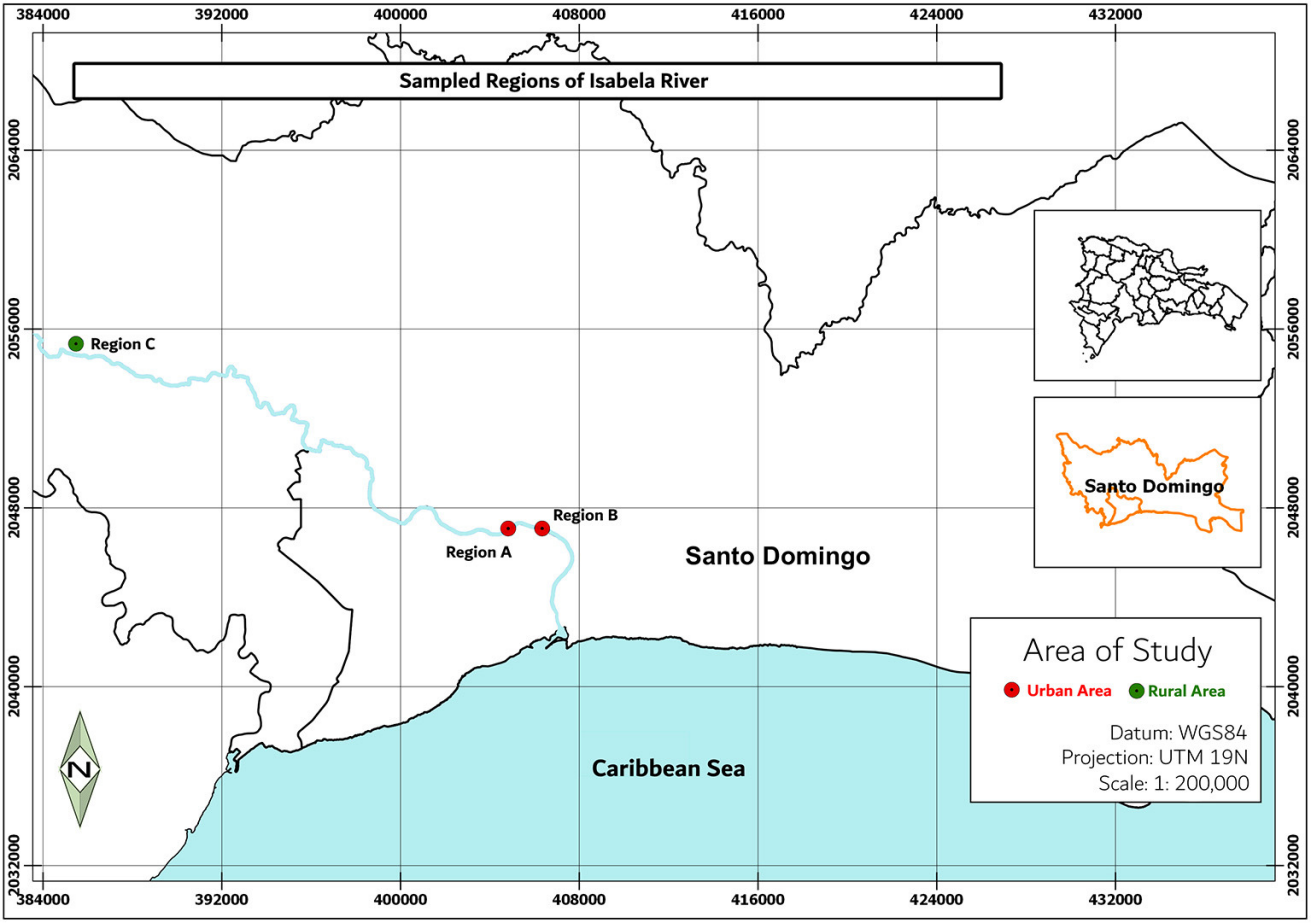
Sampling sites in the Isabela river. Region A is located near four populated areas in Santo Domingo city, region B is located at the confluence with the Ozama River, and region C is located nears the river source.

Water samples were collected from two regions of the Isabela river: a rural area with sparsely distributed residential zones and small-scale farming activities (Villa Altagracia) and a densely populated urban area (City of Santo Domingo) (Figure 1). Locations marked were selected for this study due to their immediate environments: site A (18°30′36.4″N 69°54′47.2″W) has numerous effluents such as sewage, streams, canals, and public latrines coming directly from communities of this area; site B (18°30′49.7″N 69°53′55.2″W) is near the confluence with the Ozama River; at this region, the river carries waste and pollutants produced by impoverished neighborhoods and industries located on its river banks; site C (18°41′47.9″N 70°09′31.9″W) is a rural area characterized by the presence of homesteads (Ministério de Médio Ambiente, 2012) whose activities envelop subsistence agriculture.

From each sampling site, 2 L of surface water (1 m) was collected in sterile polypropylene bottles and kept at −2°C. These samples were utilized for bacterial isolation and water quality measurements.

Temperature and pH were measured *in situ* with a multi-parameter probe (Chek-Mite pH-20; cat.: Z543047). Turbidity, phosphorus, nitrogen, and chemical oxygen demand (COD) were determined through UV spectrophotometry. Biochemical oxygen demand (BOD) was determined through barometry (BOD Track II; cat.: 2952400) by the Environmental Chemistry Laboratory of Instituto Tecnológico de Santo Domingo (INTEC). Results were evaluated according to the Dominican Environmental Norm of water Quality and Discharge.

### 2.2. Bacterial Culture and Isolation

Aliquots of 1, 10, and 50 mL were vacuum-filtered in triplicates through a 0.22 μm pore size nitrocellulose membrane (Simsii, INC) as recommended by Freitas et al. (2019). Bacteria retained in membranes were grown in two culture media: (a) MacConkey agar supplemented with imipenem (IMP) (4 μg/mL); and (b) MacConkey supplemented with cefotaxime (CTX) (8 μg/mL). Samples were incubated at 37° C from 16 up to 48 h. Isolates were obtained using the streak plate method on chromogenic culture media (ChromAgar™, France). Pure isolates were stored in 25% glycerol at −70° C.

### 2.3. Bruker BioTyper Bacterial Classification and Identification

Microbial identification was performed through MALDI-TOF (matrix-assisted laser desorption ionization-time of flight) as recommended by the CLSI guidelines (Weinstein, 2019) and (Strejcek et al., 2018) with some adaptations. To ensure reproducible results by the BioTyper® 3.1 software (Bruker Daltonics, Germany) equipped with MBT 6903 MPS library (released in 2019), MALDI BioTyper Preprocessing Standard Method and the MALDI Biotyper MSP Identification Standard Method adjusted by the manufacturer were used. Isolated colonies were cultured on blood agar for 24 h at 35°C. Approximately 0.1 mg of each new culture was inoculated in a sample carrier with the complete cell transfer protocol. Samples were coated with 1 μL of matrix solution (10 mg/mL) consisting of α-cyano-4-hydroxycinnamic acid in 50% acetonitrile and 2.5% trichloroacetic acid and left to dry at 25°C for 20 min. Identification was done in triplicates.

### 2.4. Minimum Inhibitory Concentration (MIC) Test

Isolate susceptibility testing from minimum inhibitory concentration (MIC) was conducted with the Phoenix™ M50 DB platform and classified according to the recommendations of the Clinical and Laboratory Standards Institute (CLSI) (Weinstein, 2019). The Phoenix ID broth was inoculated with isolates from a pure culture adjusted from 0.25 to 0.5 McFarland and the control strain for direct comparison, we utilized *E. coli* ATTC 25922. The suspension was poured into the susceptibility panel and loaded to the Phoenix M50, in which kinetic measurements of colorimetric and fluorimetric signals were collected every 20 min. To ensure the inoculates' purity, suspensions were subcultured on Tryptic Soy Broth (TSB) supplemented with 5% sheep blood to watch for contamination. The following antibiotics were tested: ampicillin, amikacin, amoxicillin-clavulanic acid, CTX, ceftazidime, cefuroxime, ciprofloxacin, gentamicin, trimethoprim-sulfamethoxazole, IMP, meropenem, and ertapenem.

### 2.5. Genomic DNA Extraction From Isolates

Genomic DNA was extracted from colonies incubated in TSB for 24 h at 35° C. One aliquot of 4 mL of culture was centrifuged at 8,000 *g* for 2 min. Cell pellet was subjected to the QIAamp DNA extraction kit (Qiagen, Germany) with the adaptations indicated next: the bacterial pellet was suspended in 420 μL of the modified lysis buffer (20 μL proteinase K, 200 μL of TSB, and 100 μL of Qiagen's ATL buffer), and incubated for 10 min at 56° C. The addition of 50 μL of absolute ethanol followed by 3 min incubation at room temperature concluded the adaptations; from this point in the process, the protocol continued according to the manufacturer's recommendations. DNA obtained was suspended in 50 μL of Qiagen's TE buffer. The integrity of extracted DNA was evaluated in 1% agarose gels stained with SYBR Green and ran at 100 V for 60 min.

### 2.6. Antibiotic Resistance Genes Detection by PCR Amplification

Amplification of β-lactamase genes by polymerase chain reaction (PCR) was used to determine the presence of ARGs in bacterial isolates. The strain *E. coli* ATCC 25922 was utilized as negative control. Positive controls included *E. coli* ATCC 35218 (TEM), *K. pneumoniae* ATCC 700603 (OXA and SHV), and *K. pneumoniae* ATCC BAA-1705 (KPC, TEM, and SHV). The following genes were amplified: bla_TEM_, bla_SHV_, bla_VIM_, bla_OXA_, bla_IMP_, and bla_KPC_ as recommended by Chouchani et al. (2013) and Tacão et al. (2012). Primers for these genes were previously described by Decré et al. (2010). Three multiplex PCR reactions were carried out in a final volume of 50 μL. For each reaction, 20 ng of genomic DNAs were amplified with a Promega PCR Master Mix utilizing each primer at 0.5 pg/μL. Configurations for the ABI 7500 thermocyclers utilized were as follows: initial 95°C for 5 min; 35 cycles amplification of 95°C for 60 s, 60°C for 60 s, and 72°C for 60 s; after cycles, 72°C for 10 min. Amplicons obtained from these reactions were visualized on a 1.2% agarose gel running for 90 min at 80 V to ensure separation. The molecular weight marker used was 100 bp *TrackIt* ladder by Invitrogen.

### 2.7. Physico-Chemical and Microbiological Data Analysis

Physico-chemical parameters were compared to national standards to detected discrepancies in the use of sampled water as a resource. Data were analyzed with decomposition clustering algorithms to identify similarities between sampling sites. Relative abundances were taken for analysis as the ratio between the absolute count of each measure and the total samples for such measure. For this purpose, a Python 3.7.1 Jupyter Notebooks was developed and can be accessed at GitHub (https://github.com/VictorCalderon/Isabela_Resistance).

### 2.8. Genome Sequencing, Assembly, and Analysis

Fifteen multi-drug-resistant isolates were selected according to genes identified by PCR and their clinical relevance were sequenced (Table 1).

**Table 1:** List of the sequenced genomes.

**ID**	**Identification**	**ID**	**Identification**	**ID**	**Identification**
**AI6**	*A. baumannii*	**AI12**	*A. baumannii*	**AC6**	*E. coli*
**AI10**	*A. baumannii*	**BI15**	*A. baumannii*	**AC9**	*E. coli*
**AI11**	*A. baumannii*	**BC5**	*E. cloacae*	**BI10**	*E. coli*
**BI5**	*A. baumannii*	**BI4**	*E. kobei*	**BC4**	*E. coli*
**BI9**	*A. baumannii*	**AC1**	*E. coli*	**BC8**	*E. coli*

The extraction process was previously described in “Genomic DNA Extraction from Isolates.” For the construction of sequencing libraries, (I) the genomic DNA was randomly fragmented by sonication; (II) DNA fragments were end polished, A-tailed, and ligated with the full-length adapters of Illumina sequencing, and followed by further PCR amplification with P5 and indexed P7 oligos; and (III) the PCR products as the final construction of the libraries were purified with AMPure XP system (Beckman Coulter Inc., Indianapolis, IN, USA). Sequencing library size distribution quality control was performed with an Agilent 2100 Bioanalyzer (Agilent Technologies, CA, USA) and quantified by real-time PCR (to meet the criteria of 3 nM). Whole genomes were sequenced using Illumina NovaSeq 600 using the PE 150 strategy at the America Novogene Bioinformatics Technology Co., Ltd.

Genomes were assembled using the Assembly HiSeq Pipeline, a SnakeMake pipeline to assemble sequencing data produced by Illumina (Miranda and Ramos, 2020). The pipeline integrates different quality control tools like FastQC (Andrews, 2010) to analyze and visualize read quality, AdapterRemoval v2 (Schubert et al., 2016) for removing sequencing adapters, and KmerStream (Melsted and Halldórsson, 2014) for computing k-mer distribution. For the genome graph construction, two main assemblers were used: Edena V3 (Hernandez et al., 2008) and Spades 3.9.1 (Bankevich et al., 2012); CD-HIT (Fu et al., 2012) and Unicycler (Wick et al., 2017) were used to optimize and integrate the assemblies previously produced. Whole-genome annotation was performed with RAST (Aziz et al., 2008) and Prokka (Seemann, 2014). To predict and reconstruct individual plasmid sequences in the genome assemblies, we used MOB-recon (Robertson and Nash, 2018). Finally, QUAST (Gurevich et al., 2013) computed assembly quality metrics and each individual genome phylogenetic affiliation was confirmed through JSpeciesWS web tools (Richter et al., 2016) using the contigs generated by the assemblies. The whole genomes shotgun projects have been deposited to DDBJ/ENA/GenBank. Table 3 contains the main characteristics of the genomes.

Bioinformatic analyzes of the multi-resistant genomes started with Resistance Genes Identifier (RGI) with the CARD protein database (Alcock et al., 2020) and ResFinder-4.0 (Bortolaia et al., 2020) to predict the resistance genes. Plasmid detection was conducted through the MOB-suite (Robertson and Nash, 2018) and PlasmidFinder-2.1 (Carattoli et al., 2014). For pathogenicity classification of each of the strains, PathogenFinder-1.1 (Cosentino et al., 2013) was utilized. VirulenceFinder-2.0 (Kleinheinz et al., 2014) was used to determine the virulence factors of each genome. The serotypes of the *E. coli* genomes were determined using SerotypeFinder-2.0 (Joensen et al., 2015), and the number of mobile elements were determined by MobileElementFinder (Johansson et al., 2020).

## 3. Results

### 3.1. Physico-Chemical and Microbiological Results

Physico-chemical parameters of sampled sites were mostly within Dominican regulations for Water Quality and Discharges from 2012 (Supplementary Table 1) (Ministério de Médio Ambiente, 2012). Waters in sites A and B were classified as superficial water (class C) based on the previously mentioned norm. These waters can be used for navigation, cooling, and activities that do not imply direct contact. These regulations are not enforced since many residents of neighboring communities consume these waters for recreation and ingestion.

Sampling site C had slightly higher (≥2 mg/L) levels of biological oxygen demand (BOD); this could suggest a greater abundance of microorganisms. Region C was classified by the Dominican norm as a class B superficial water: this classification allows for consumption after treatment and is mostly used for agriculture and aquaculture.

Dispersion analyses suggest grouping water samples in two main categories based on significant variations found in the distribution of BOD, dissolved oxygen, and turbidity. According to this classification (Supplementary Figures 1, 2), sites A and B present a similar enough distribution of physico-chemical parameters to classify them in the same group by a hierarchical clustering algorithm based on standardized Euclidean distances. Site C, on the contrary, clustered differently. To assess the quality of formed clusters, a PERMANOVA test was performed demonstrating statistical difference (*p* ≤ 0.028) between groups.

### 3.2. Isolates Identification and Characterization

A total of 55 bacteria from the Isabela river were isolated and stored. From those bacteria, 27 isolates were recovered from the culture media supplemented with CTX and 28 were recovered from media supplemented with IMP. From site A, there were a total of 17 isolates: 9 from CTX media and 8 from IMP media. Site B presented 24 isolates: 8 from CTX media and 16 from IMP media. Site C has 14 isolates: 10 and 4 from CTX and IMP media, respectively. Isolate identification along with MALDI-TOF similarity score can be found in Supplementary Table 2.

**Figure 2:**
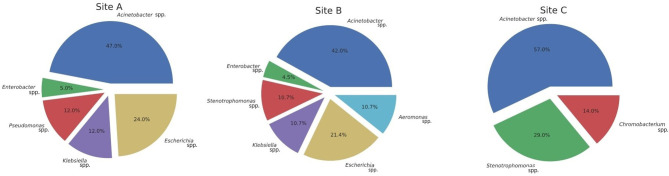
Distribution of isolates.

MALDI-TOF mass spectrometry analyses of ribosomal protein composition revealed a total of 8 different genera (Figure 2). Most common genera for Gammaproteobacteria were as follows: *Acinetobacter* (44.6%), *Escherichia* (18%), *Stenotrophomonas* (12.5 %), *Pseudomonas* (7.1%), *Enterobacter* (5.3%), *Klebsiella* (3.5%), and *Aeromonas* (3.5%). Only *Chromobacterium* (3.5%) from the class Betaproteobacteria was isolated. In CTX culture media, *Acinetobacter* and *Escherichia* were the most common genera. From IMP media, *Acinetobacter* and *Stenotrophomonas* were the most abundant. *Chromobacterium* was only retrieved from the CTX media. Supplementary Table 2 contains complete MALDI-TOF results.

### 3.3. Antibiotic Susceptibility Profiles and ARGs on Clinically Relevant Isolates

For the antibiotic susceptibility profiles and resistomes analysis, we selected 20 isolates that are classified as pathogenic and that may represent a risk to human health according to World Health Organization (Tacconelli et al., 2017).

**Figure 3:**
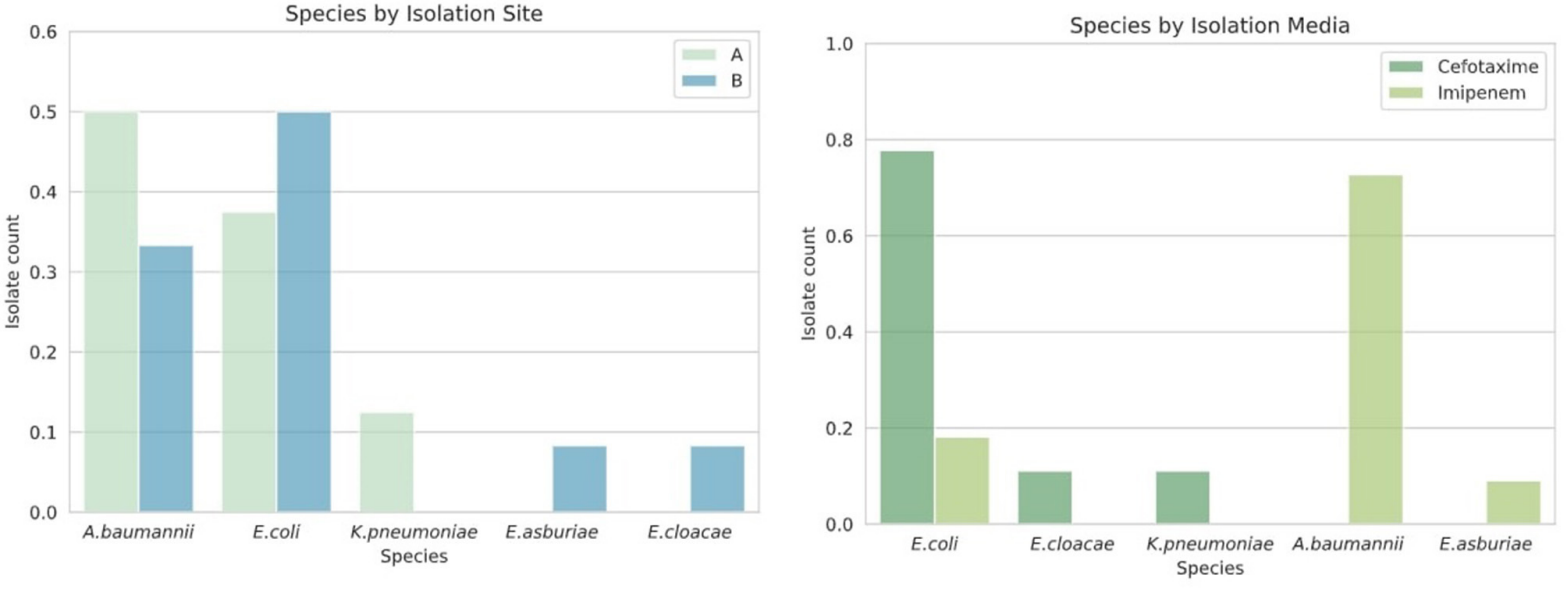
Relative abundance of clinically important bacteria by sampling site. Other genera were isolated by this method, and only Extended Spectrum β-Lactamases (ESBLs) positive bacteria were included.

Eight resistant isolates from three different genera were obtained from site A as represented in Figure 3. These genera included *Acinetobaceter* (50%), *Escherichia* (37.5%), and *Klebsiella* (12.5%). Bacteria isolated from media supplemented with IMP were ubiquitously resistant to meropenem (100%) in site A. From this same site, bacteria isolated from CTX supplemented media presented resistance to amoxicillin, cefazolin, cefepime, ceftriaxone, cefuroxime, and ciprofloxacin, the results can be seen in Table 1. Supplementary Table 3 contains complete MIC resistance sresults.

**Figure 4:**
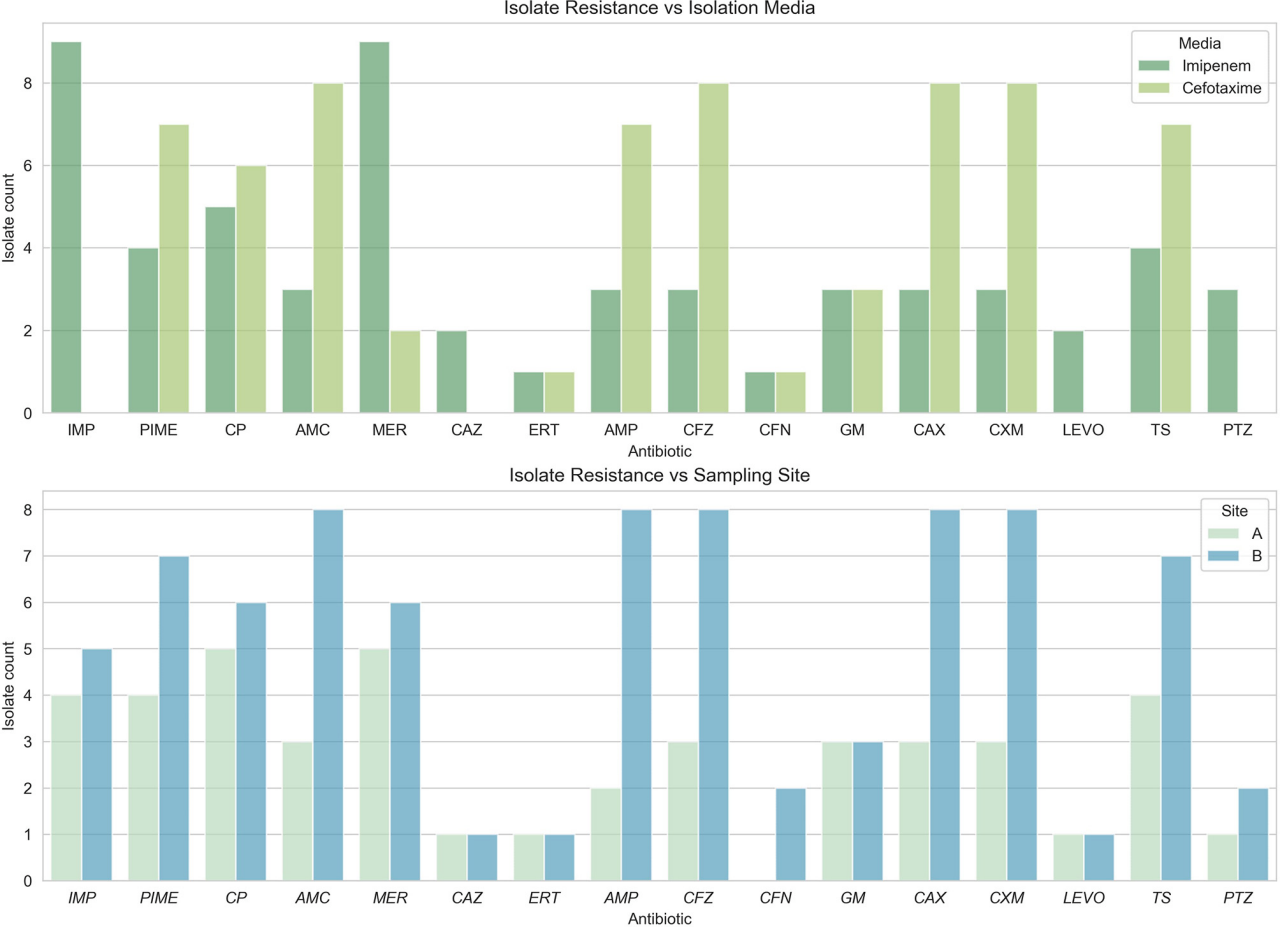
Resistance phenotypes found per sampling site. All antibiotics tested presented resistance in the samples obtained. **(Top)** Phenotype by sites; **(Bottom)** Phenotype by media.

Twelve resistant isolates from genera *Escherichia, Acinetobacter*, and *Enterobacter* were obtained from region B. All isolates from IMP-supplemented media presented resistance to meropenem, cefuroxime, and ciprofloxacin. All isolates from media supplemented with CTX were resistant to amoxicillin, ampicillin, cefazolin, ceftriaxone, and cefuroxime. Only 75% of these isolates also exhibited resistance to cefepime and trimethoprim-sulfamethoxazole (Figure 4).

Region C isolates belonged to *Stenotrophomonas* and *Chromobacterium* genera. *Acinetobacter* were also found in this region, but double synergy disc assays (Kaur et al., 2013) discarded the possibility of active ESBLs ARGs.

Sixty-five percent (65%) of all isolates exhibited multi-resistant phenotype under the CLSI classification standards (Table 2) (Weinstein, 2019). *Enterobacteria* isolated presented the most diverse phenotypic resistance, demonstrating resistance to at least three families of antibiotics. *Enterobacter kobei* presented diverse resistance profiles, exhibiting resistance to 11 antibiotics from five different families. Multi-resistant *Klebsiella pneumoniae* obtained were resistant to carbapenems.

**Table 2:** Resistance phenotype from minimum inhibitory concentration (MIC) and resistance genes detected through the polymerase chain reaction (PCR) technique.

**ID**	**Identification**	**Resistance Phenotype**	**PCR+**
AI6	*A. baumannii*	CAX, IMP, MER, PTZ	
AI10	*A. baumannii*	CAX, PIME, IMP, MER,PTZ	
AI11	*A. baumannii*	PIME, CAX, CP, GM, IMP, MER, PTZ, TS, CAZ, LEVO	
AI12	*A. baumannii*	CAX, IMP, MER	
AC1	*E. coli*	CP, ERT, MER	OXA
AC6	*E. coli*	AMC, CFZ, PIME, CAX, CXM, CP, GM, TS	TEM
AC8	*K. pneumoniae*	PIME, CAX, CXM, TS	SHV
AC9	*E. coli*	AMP, CFZ, PIME, CAX, CXM, CP, GM, TS	
BI3	*E. coli*	AMP, CFZ, PIME, CAX, CXM, CP, TS	TEM
BI4	*E. kobei*	AMC, CFZ, CFN, CAX, CXM, CP, ERT, IMP, MER, PTZ	KPC
BI5	*A. baumannii*	CAX, IMP, MER	
BI9	*A. baumannii*	CAX, IMP, MER	
BI10	*E. coli*	AMP, CFZ, PIME, CAX, CXM, CP, GM,TS	OXA
BI12	*A. baumannii*	PIME, CAX, CP, GM, IMP, MER, PTZ, TS, CAZ, LEVO	
BI15	*A. baumannii*	CAX, IMP, MER	
BC3	*E. coli*	AMP, CFZ, PIME, CAX, CXM, TS	TEM
BC4	*E. coli*	AMP, CFZ, PIME, CAX, CXM, TS	TEM
BC5	*E. cloacae*	AMC, AMP, CFZ, PIME, CFN, CAX, CXM	
BC6	*E. coli*	AMP, CFZ, PIME, CAX, CXM, CP, TS	
BC8	*E. coli*	AMP, CFZ, PIME, CAX, CXM, ERT, GM, MER, TS	TEM

**Figure 5:**
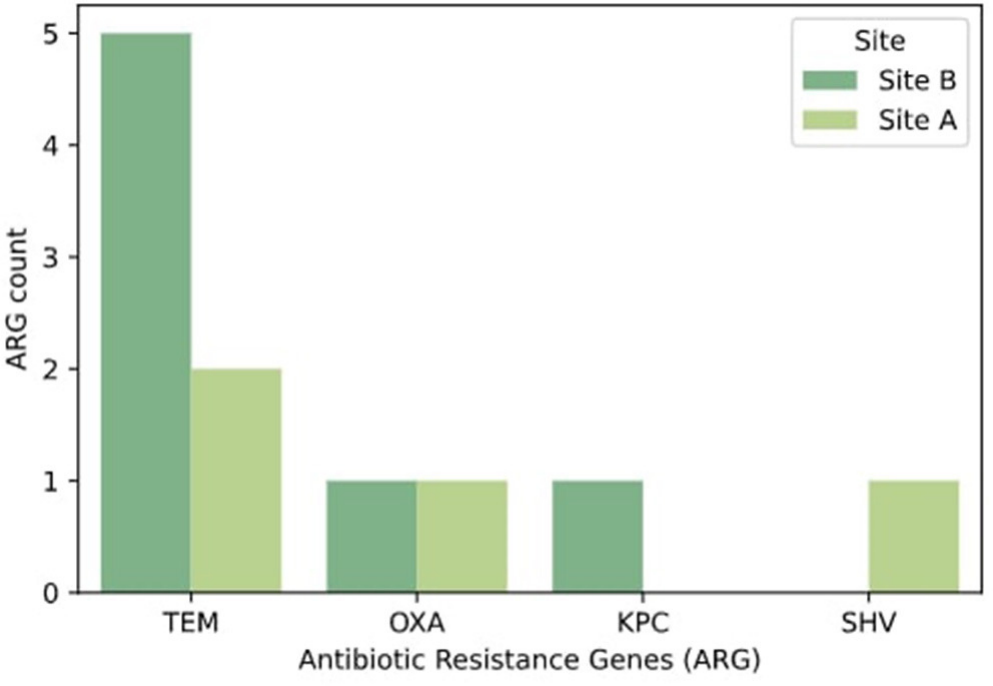
Relative abundance of extended spectrum β-lactamases identified by sampling site. Only positive samples by PCR amplification were selected for this visualization.

Twenty-five percent (*n* = 5) of DNA samples extracted from all isolates were amplified by primers for *bla*_*TEM*_ genes, mostly from enterobacteria (Figure 5). *bla*_*OXA*_ genes were found in *E. coli* (*n* = 2), representing 10% of all samples. *bla*_*SHV*_ genes were determined in 5% of sampled bacteria and *bla*_*KPC*_ (*n* = 1) was observed in one *E. kobei* isolate.

### 3.4. Analysis of Multi-Resistance Genomes

**Figure 6:**
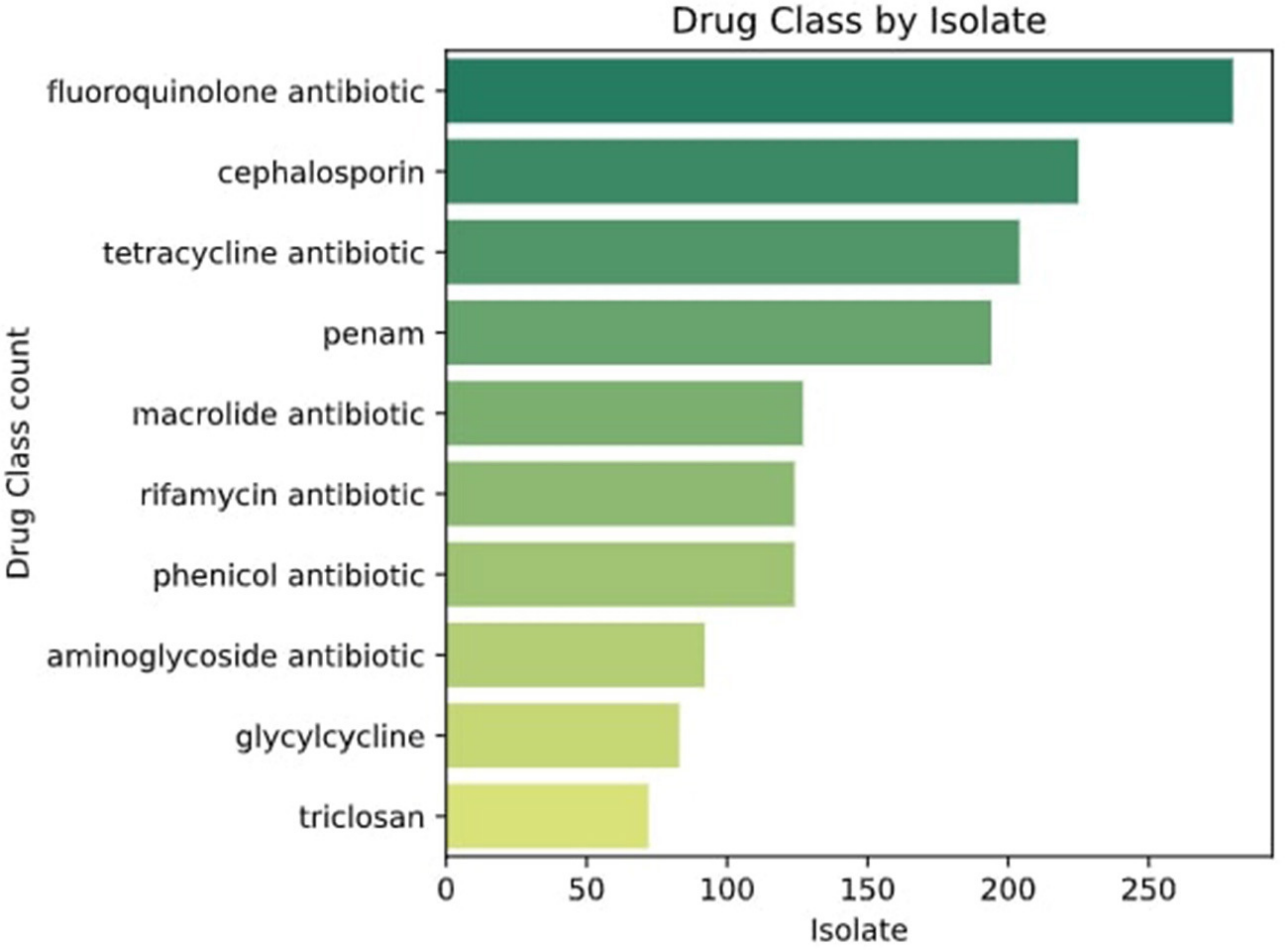
Classes of drugs to which genomes are resistant according to CARD and ResFinder.

To confirm the resistomes identified through PCR, 15 multi-resistant genomes were sequenced. The genomes were selected considering the number of resistance genes detected and the diversity of bacteria. The species were confirmed through taxonomy using JSpeciesWS (Richter et al., 2016). Resistance Genes Identifier (RGI) results from the CARD tool (Alcock et al., 2020) with a perfect protein sequence score (100%) were selected. The genomes sequenced displayed resistance to fluoroquinolones, penicillins, tetracyclines, cephalosporins, and phenicols among other families of antibiotics (Figure 6).

#### 3.4.1. *Escherichia coli*

A total of six *Escherichia* isolates were sequenced. Several parameters were computed for each isolate, among them were genome size, GC% content, and total predicted coding sequences (CDS). *Escherichia* isolates sequenced presented an average genome size of 4.7 Mb, except for isolate BC6 that presented approximately 6 Mb of total genomic length. All *Escherichia* isolates had an average GC% content of 50 as seen in Table 3.

**Table 3:** Major genomic characteristics of genomes obtained from Isabela river.

**Genome**	**Accession**	**%GC**	**CDS**	**N50**	**Size (pb)**	**Plasmids**
***Enterobacter cloacae*** ***INTEC_BC5_1.1***	JACSEN000000000	54.9	4,877	266411	4,997,895	pK245,pECL_A
***Enterobacter kobei*** ***INTEC_BI4_1.1***	JACSEP000000000	54.2	5,599	127742	5,411,162	pHAD28, pIGMS32, pCAV1099-114, pKPC_CAV1193
***Escherichia coli*** ***INTEC_AC6_1.1***	JACSHL000000000	50.8	4,791	113960	4,705,045	plasmid_F
***Escherichia coli*** ***INTEC_BI10_1.1***	JACSEO000000000	50.6	5,867	125667	4,838,800	plasmid_F
*Escherichia coli* *INTEC_AC1*	SAMN16191447	50.8	4,862	125825	4,852,839	unnamed_plasmid
*Escherichia coli* *INTEC_AC9*	SAMN16191377	50.8	4,620	113902	4,706,597	unnamed_plasmid
***Escherichia coli*** ***INTEC_BC4***	JACZEG000000000	50.9	4,867	86475	4,965,451	pKPN-IT, unnamed_plasmid
***Escherichia coli*** ***INTEC_BC8***	JACZEH000000000	50.9	6,113	147588	6,104,323	pHAD28, plasmid_F, pHN7A8, p_R46, pO111_2
***Acinetobacter baumannii*** ***INTEC_AI11_1.1***	JACSHM000000000	38.9	3,976	224239	3,879,719	
***Acinetobacter baumannii*** ***INTEC_BI5***	JACXKJ000000000	38.8	4,175	383375	4,461,496	
***Acinetobacter baumannii*** ***INTEC_BI9***	JACXLE000000000	38.7	3736	472074	5,145,612	
*Acinetobacter baumannii* *INTEC_AI6*	SAMN16287478	38.8	4,480	400571	4,690,858	pMG828-1
***Acinetobacter baumannii*** ***INTEC_AI12***	JACZEF000000000	38.7	3,733	471980	5,092,223	
***Acinetobacter baumannii*** ***INTEC_AI10***	JACZEE000000000	38.8	4,204	372323	4,491,903	
***Acinetobacter baumannii*** ***INTEC_BI15***	JACZEI000000000	38.8	4,233	383375	4,461,496	

Between 4,600 and 6,100 CDS were predicted for these isolates, where approximately 1% of these genes were related to virulence, disease, and defense. Among genes found in these sequences were beta-lactamase resistance alleles CTX-M-15, CTX-M-55, TEM-1, OXA-1, OXA-72, ampC, ampC1, and ampH with a perfect protein score (Table 4). These isolates also presented other ARGs with resistance mechanisms of antibiotic efflux, inactivation, target alteration, and target replacement.

**Table 4:** Resistance genes identified and annotated by CARD and/or ResFinder.

**Genome**	**Resistance genes**	**Resistance mechanism // Drug class**
*Escherichia coli* *[-0.3pt] INTEC_AC6*	msbA, mdtN, mdtP, cpxA,kdpE, emrB, emrR, AcrF, [-0.3pt] AcrE, AcrS, mdtE, mdtFgadX, emrY, emrK, evgA, [-0.3pt] TolC, H-NS, mdtH, mdtG, acrA, acrB, YojI, qacEdelta1, marA	Antibiotic efflux
	ampC, ampH, CTX-M-55, aadA, aadA2, mphA, TEM-1	Antibiotic inactivation
	PmrF	Antibiotic target alteration
	sul3,dfrA12, sul1	Antibiotic target replacement
*Enterobacter cloacae* *[-0.3pt] INTEC_BC5*	QnrS1	Quinolone target protection
	CTX-M-15	*B*-lactamase inactivation
*Acinetobacter* *[-0.3pt] baumannii* *[-0.3pt] INTEC_AI11*	adeL, abeS, adeK,adeI	Antibiotic efflux
	OXA-132, OXA-72	*B*-lactamase inactivation
	sul2	Sulfonamide target replacement
*Escherichia coli* *[-0.3pt] INTEC_BI10*	evgA, TolC, acrB, acrA, AcrE, AcrS,mdtG, cpxA,mdtE, msbA, [-0.3pt] TolC, H-NS,marA, emrB, emrR, qacEdelta1, mdtA, mdtB, emrR	Antibiotic efflux
	aadA5, CTX-M-15,mphA, AAC(6')-Ib-cr, OXA-1	Antibiotic inactivation
	sul1	Sulfonamide target replacement
*Enterobacter kobei* *[-0.3pt] INTEC_BI4*	KPC-3	Carbapenem inactivation
	QnrS1, QnrB19	Quinolone target protection
*Escherichia coli* *[-0.3pt] INTEC_AC1*	emrB, emrA, baeR, baeS, H-NS, mdtG, evgS, evgA, emrY, cpxA, [-0.3pt] kdpE, mdtP, mdtN, mdtE, mdtF,gadX, TolC, msbA,acrD,YojI, [-0.3pt] baeR,baeS,cpxA, qacEdelta1, acrB, acrA, baeR, baeS, acrD, marA	Antibiotic efflux
	ampC, ampC1, CTX-M-15, aadA5, mphA, OXA-1, ampH	Antibiotic inactivation
	PmrF, bacA	Antibiotic target alteration
	sul2, dfrA17, sul1	Antibiotic target replacement
*Escherichia coli* *[-0.3pt] INTEC_AC9*	TolC, msbA, mdfA, mdtN, mdtO, mdtP, cpxA, rsmA, emrB, emrA, [-0.3pt] emrR, AcrF, AcrE, AcrS,KpnE, KpnF, mdtE, mdtF, gadX, mdtA, [-0.3pt] mdtB,mdtC, baeS, mdtM,acrD, evgS, evgA, emrK, emrY,mdtG, mdtH, [-0.3pt] H-NS, marA	Antibiotic efflux
	ampC, ampC1, ampH	Ampicilin inactivation
	bacA, eptA, PmrF	Antibiotic target alteration
*Acinetobacter baumannii* *[-0.3pt] INTEC_BI5*	adeL, abeS, adeK, AbaQ	Antibiotic efflux
	OXA-72	*B*-lactamase inactivation
*Acinetobacter baumannii* *[-0.3pt] INTEC_BI9*	adeL, abeS, adeK, AbaQ	Antibiotic efflux
	OXA-72	*B*-lactamase inactivation
*Escherichia coli* *[-0.3pt] INTEC_BC4*	TolC, msbA, emrK, evgA, emrR,emrB, cpxA, acrA, acrB, gadXmdtF, [-0.3pt] mdtE,H-NS, AcrS, baeS, baeR, YojI, mdtG, AcrS, emrB, emrR	Antibiotic efflux
	CTX-M-15, TEM-1	*B*-lactamase inactivation
	bacA, PmrF, QnrS1	Antibiotic target alteration
	sul2, dfrA14	Antibiotic target replacement
*Escherichia coli* *[-0.3pt] INTEC_BC8*	mdtN, mdtB, baeS, baeR, emrR,msbA, acrB, acrA, cpxA,AcrE [-0.3pt] AcrS, TolC, evgS, evgA, emrK,mdtG, mdtH, H-NS, marA	Antibiotic efflux
	linG, FosA3, CTX-M-55,AAC(3)-IId	*B*-lactamase inactivation
	PmrF, bacA	Antibiotic target alteration
	QnrB19	Antibiotic target protection
	sul2	Antibiotic target replacement
*Acinetobacter baumannii* *[-0.3pt] INTEC_AI12*	AbaQ, abeS, adeL, adeK	Antibiotic efflux
	OXA-72	*B*-lactamase inactivation
*Acinetobacter baumannii* *[-0.3pt] INTEC_AI6*	AbaQ,adeL, abeS, adeK, adeI	Antibiotic efflux
	OXA-72	*B*-lactamase inactivation
*Acinetobacter baumannii* *[-0.3pt] INTEC_BI15*	AbaQ, abeS, adeL, adeK	Antibiotic efflux
	OXA-72	*B*-lactamase inactivation
*Acinetobacter baumannii* *[-0.3pt] INTEC_AI10*	AbaQ, abeS, adeL, adeK	Antibiotic efflux
	OXA-72	*B*-lactamase inactivation

*Continue-resistance genes identified and annotated by CARD and/or ResFinder*.

According to Pathogen-Finder-1.1, these genomes had 92% probability of being human pathogens. Among virulence factors found in these isolates were *cma, hlyF, iss, iucC, iutA, ompT, sitA, terC*, and *traT*. Inc plasmidic sequences were also found in these genomes corresponding to IncFIB(AP001918) (682 bp), IncFIC(FII) (449), and IncY (765). Serotype classification for these genomes were O101, O162, H10, H9, O8, and H4 (Table 5).

**Table 5:** Results of the different analyzes performed on the genomes.

**Genome**	**Probability of being a human pathogen**	**Virulence factors**	**Serotype**	**Multilocus sequence typing (MLST)**
*Enterobacter cloacae* *INTEC_BC5_1.1*	76.2%	*terC*	–	ST:976
*Enterobacter kobei* *INTEC_BI4_1.1*	76.7%	*ccl,fyuA, irp2, terC*	–	*ST:99*
*Escherichia coli* *INTEC_AC6_1.1*	93.1%	*cma,cvaC,gad,hlyF, iss,iucC,iutA,ompT,* *sitA,terC,traT*	O101, H10	ST:10
*Escherichia coli* *INTEC_BI10_1.1*	93.8%	*fyrA,gad,irp2,iucC,iutA,* *ipfA,ompT,sitA,terC*	H9	ST:410
*Escherichia coli* *INTEC_AC1*	92.9%	*etsC, gad, * *papC, terC, traT*	O101, H10	ST:10
*Escherichia coli* *INTEC_AC9*	93.3%	*cma, cma, cvaC,gad,hlyF,iss,* *iucC,iutA,ompT,sitA,terC,* *traT*	O101,H10	ST:10
*Escherichia coli* *INTEC_BC4*	93.1%	*gad,iss,terC*	O8,H4	ST:46
Escherichia coli INTEC_BC8	92.7%		H10	ST:2705
*Acinetobacter baumannii* *INTEC_AI11_1.1*	85.9%	-	–	ST:1635
*Acinetobacter baumannii* *INTEC_BI5*	86%	-	–	ST:46
*Acinetobacter baumannii* *INTEC_BI9*	86.1%	-	–	Nearest ST:1195
*Acinetobacter baumannii* *INTEC_AI6*	85.9%	-	–	Nearest ST:1488
*Acinetobacter baumannii* *INTEC_AI12*	86.1%	-	–	Nearest ST:1195
*Acinetobacter baumannii* *INTEC_AI10*	86%	-	–	Nearest ST:1195
Acinetobacter baumannii INTEC_BI15	86%	-	–	Nearest ST:1195

#### 3.4.2. *Acinetobacter baumannii*

A total of seven *Acinetobacter baumannii* isolates were sequenced. Draft genome sizes for these isolates ranged from 3.8 to 5.8 Mb. All *A. baumannii* isolates had an average GC% content of 38.85 as seen in Table 3. Between 3,900 and 4,500 CDS were predicted for each genome, with 19 CDS related to virulence, disease, and defense. No virulence factors were found in these genomes by Virulence-Finder2.0. Complete beta-lactam resistance genes alleles sequences found in these genomes were OXA-132 and OXA-72. These genes presented resistant mechanisms of antibiotic inactivation (Table 4). According to Pathogen-Finder1.1, these isolates can infect humans with a probability of 86%. These isolates did not contain any mobile genetic element (MGE).

#### 3.4.3. *Enterobacter cloacae*

In the INTEC BC5 genome, 4,993 CDS were identified, 58 CDS participating in virulence, disease, and defense processes and 36 in antibiotic resistance and toxic compounds. This genome has a GC% of 54.87, an N50 of 266411, and a total length of 5,169,767 bp. Plasmids related to antibiotic resistance such as IncFIB (pECLA) and IncR were found in this genome (Table 3). Genes QnrS1, dfrA14, and CTX-M-15 were identified in this bacterium's resistome by RGI (Table 4). Analysis performed by VirulenceFinder reveal the presence of terC, a virulence factor correlated with more severe infections. This genome has a 76.2% probability of being a human pathogen. Analysis done through PathogenFinder matched 70 families affiliated with pathogenicity (Table 5).

#### 3.4.4. *Enterobacter kobei*

This genome has a size of 5,761,677 pb, and GC% of 54. RAST identified 5,423 CDS, of which 71 CDS (1.3%) are related to virulence, disease, and defense, and 41 (0.71%) of these CDS are related to resistance to antibiotics and toxic compounds. The plasmids IncFIB(K)(pCAV1099-114), pKPC-CAV1193, and pKPC-CAV132 were found inside this genome (Table 3). We confirm three resistance genes (KPC-3, QnrS1, and QnrB19, blaACT-9) in this genome that use antibiotic inactivation and antibiotic target protection as the primary resistance mechanisms according to CARD and resFinder tools. According to PathogenFinder, this genome has a 77% probability of being human pathogenic (Table 5).

## 4. Discussion

This research presents the first evaluation of environmental ARGs in the Dominican Republic, specifically in the Isabela river. This river has been essential for the development of the city of Santo Domingo since colonial times, being one of the most important navigable rivers and water sources of Santo Domingo city. Since the 1970s, this river's water quality has been suffering due to pollution caused by unplanned urban growth. The situation is exacerbated by the presence of new industries, farms, and abattoirs on its margins and the absence of proper environmental regulations (Chantada, 1991). These conditions have been previously described as altering factors of resistomes and microbiomes of lotic environments like the Isabela river. Perturbation in these environments can result in higher prevalence of antibiotic-resistant bacteria and resistance genes; these modifications may pose a significant danger to human and environmental health. This study allowed for the exploration and description of the resistant microbial composition present in three sites of the Isabela river, with particular focus on genes associated with β-lactamase-producing Gram-negative bacteria of clinical relevance.

### 4.1. Analysis of the Physico-Chemical and Microbiological

Most physico-chemical parameters examined from samples collected were within the Dominican Standard Surface Waters Norm (Ministério de Médio Ambiente, 2012). However, BOD and COD values at sites A and B were measured higher than those at site C. This might suggest the presence of large amounts of organic material in sites A and B; similar values were observed near site A by Gutiérrez (2014). Elevated nitrogen and turbidity measurements observed in points A and B might indicate the presence of human pollutants in the water. Sites A and B were observed with approximately twice coliform activity (CFU) compared to site C. High coliform activity has been previously described in these sites by Gutiérrez (2014) and Emmanuel and Clayton (2019) as a result of wastewaters discharge from public latrines, industries, and hospital among other; this is considered the main problem affecting the river's health.

In a recent study published by Mart́ınez et al. (2019), only 18% of Santo Domingo's wastewaters are treated before discharge into important tributaries; the other 82% which come from the city's public latrines, hospitals, and industries reach the Caribbean sea and the main rivers practically untreated. This situation affects water quality significantly as determined by the eutrophication of the Isabela river experiments with the appearance of *Eichhornia crassipes* and algae of the genera *Anabaena, Tabellar*í*a*, and *Asterionella*. These species and genera of algae are characteristic of polluted waters and have been described as altering factors of bacterial communities present in the lotic environments (Bertness MD, 2002; Gutiérrez, 2014).

### 4.2. Strains Resistant to Antibiotics and ARGs Analysis

In our research, we identified and isolated four bacterial species that presented multi-resistance as described by the CLSI (Weinstein, 2019). These isolates are included in the World Health Organization's list of multi-resistant pathogenic bacteria of higher risk to human health (Tacconelli et al., 2017). We also described and isolated carbapenem resistant *Enterobacteriaceae* and *Acinetobacter* as also observed by Tacconelli et al. (2017). The studies of Espinal et al. (1993) and Gutiérrez (2014) also detected the presence of these bacteria in the river bed, with a higher frequency close to points A and B. The prevalence of these bacteria in the river can be the result of high anthropogenic pressure the river suffers due to the populations inhabiting its banks.

Isolates identified were compared to the most common resistant bacteria in the Annual Report of the network from monitoring/surveillance of antibiotic resistance and health care associated infections-2014 (Red de Monitoreo, 2014); it was highlighted that the bacteria detected by this method usually appear in the clinical setting in the Dominican Republic. This report established *Escherichia* and *Klebsiella* as the most prevalent genera in clinical environments, both of which were isolated from river samples harboring ARGs such as bla_OXA_, bla_TEM_, and bla_SHV_. The presence of these bacteria in the river could be an indication of the existence of some transfer mechanism from the environment to the clinical environment or vice versa. More analyses are needed to determine the precedence of these pathogens.

*Enterobacteriaceae* presented the highest phenotypic diversity, being resistant to at least three families of antibiotics. *Acinetobacter* showed less phenotypic diversity, which suggests that it could have developed resistance through porin closure or efflux pumps, as described by Vila et al. (2007). *E. kobei* presented the most different resistance profile, being resistant to 11 antibiotics from five different families, and this has been previously observed by Andrade et al. (2018).

Multi-resistant *E. kobei* obtained were resistant to carbapenems. This bacterium contains the plasmid pK245, which has been associated with a quinolone resistance gene (qnrS) in Enterobacteriaceae, which was also detected in the genome. This plasmid has multiple insertion elements, facilitating disseminating of antimicrobial resistance determinants to other bacteria present in the river (Chen et al., 2006). This isolate also has a CTX-M-15 gene, which may be related to this bacterium's probability of becoming a human pathogen. Plasmids found in the genomes of *E. coli* are conjugative plasmids belonging to the IncF family, which has been related to the resistance of the genes bla_OXA_ and bla_TEM_ in Enterobacteriaceae. These plasmids are related to water contamination by human and livestock waste, as observed on the Isabela river banks, where the wastewaters arrive without any treatment. The presence of the bacteria containing these plasmids shows that river water consumption increases the risks of contamination by resistant pathogenic bacteria and favoring the transmission of resistance between humans and the environment (Shakibaie et al., 2009; Lyimo et al., 2016).

Sequencing data revealed alleles CTX-M-55 and CTX-M-15 in Dominican isolates. CTX-M genes have been associated with pathogenic bacteria of the *Enterobacteriaceae* family as well as mobile genetic elements (Cantón and Coque, 2006; Bevan et al., 2017). It has also been described in similar lotic environments by Alves et al. (2020), de Oliveira et al. (2017), Nascimento et al. (2017), and Dropa et al. (2016).

The β-lactamase bla_TEM_ gene was observed in 25% of the isolates, mostly distributed in *enterobacteriaceae*. Sequencing data revealed allele TEM-1 present in our samples, this gene has been related to families of β-lactamases that confer resistance to penicillin and to first- and second-generation cephalosporins in Gram-negative (Bradford, 2001; Alves et al., 2020). The bla_OXA_ (class D) genes found belonged to *E. coli* (n = 2), which are present in 10% of the sample. Sequencing demonstrated a variety of alleles present in our samples including OXA-132, OXA-72, and OXA-1; this gene confers resistance to ampicillin and cephalothin (Turton et al., 2006; Evans and Amyes, 2014). bla_SHV_ gene appeared in one isolate, and this gene has been related to resistance to penicillin in *K. pneumoniae* isolates (Paterson et al., 2003), and *bla*_*KPC*_ (*n* = 1) was observed in an *E. kobei* isolate, specifically a KPC-3 allele from *E. kobei*. This gene was first described by Yigit et al. (2001) and has been responsible for critical care infections in hospitals (Rodŕıguez-Zulueta et al., 2013).

Most bacteria presented a *terC* virulence factor, related to colicin resistance and phage inhibition. Bacteria that present the *ter* operon can reproduce and survive in phagocytes, which allows them to overcome one of the hosts' primary defenses for eradicating infectious pathogens (Whelan et al., 1995; Turkovicova et al., 2016). We also identified virulence factors *gad* and *iss* inside the genomes of various *E. coli* isolates. The presence of these genes may be a major component in the classification of these isolates as highly pathogenic (Chen et al., 2018).

The abundance of resistance phenotypes to ampicillin and amoxicillin in site B is more than twice that present in site A. Sites A and B are both urban areas in the middle of the city of Santo Domingo, but site A had more effluents from hospitals than site B. On the other hand, site B resides after many improvised effluents from surrounding residents, and this could help explain the rise in resistant isolates. This could possibly be explained by positive selection for these characteristics. Also, the popularity and ease of access to these same antibiotics that are currently sold without need for prescription in government pharmacies (Ortiz, 2017; Promese-cal, 2020) may have a role in this finding. In some cases, this resistance is related to the expression of the intrinsic enzyme AmpC.

Antibiotics with the least resistance found in site A were cefoxitin (CFN), levofloxacin (LEVO), and ertapenem (ERT). Antibiotics that demonstrated less resistance in our samples from site B were ceftazidime (CAZ), ertapenem (ERT), and levofloxacin (LEVO); coincidentally, these have low affordability and higher prices, which may suggest a correlation. The absence of regulations on antimicrobial sales control is of great concern to the Dominican public health system. Residents of Dominican Republic have a well-establish culture of self-medication, leading to the abuse and misuse of antibiotics (Bautista, 2018).

Our analyses and results strongly suggest the existence of anthropic action to which the Isabela river is being subjected as it runs though the city of Santo Domingo (urban sites). The behavior of inhabitants as well as the lack of infrastructure has caused the river to become a reservoir of multi-resistant bacteria and ARGs. Nevertheless, this is the first study to confirm the presence of ARGs of last resort in environmental settings of the Dominican Republic. The data were collected using culture-dependent methodologies, and only susceptibility analyses and detection of resistance genes were performed in clinically relevant bacteria, although environmental bacteria were also isolated and characterized within the study results.

All sequenced genomes in this study were deposited to publicly available databases (DDBJ/ENA/GenBank). This will allow other researchers to compare their findings to ours and identify a probable origin of the pathogen, improving the epidemiological knowledge around multi-resistant infections in the Dominican Republic.

Future studies with culture-independent techniques such as shotgun metagenomics would allow the completion of already existing data and the accomplishment of more robust analyses.

## 5. Conclusion

In this study, the presence of multi-resistant bacteria in the anthropogenically impacted Isabela river of Santo Domingo, Dominican Republic was determined. The main objective was to elucidate the relationship between resistance genes abundance in rural (low to none untreated wastewater effluents) and urban (many untreated wastewater effluents) environments to help develop strategies to prevent the spread of ARGs to the population and assess the conditions of the sampled water.

Most common genera of bacteria detected in the different sampling sites of the river were *Acinetobacter* and *Escherichia*, being the latter a frequent human pathogen listed in the antibiotic resistance surveillance report from national health authorities. The majority of clinically relevant strains (56%) isolated from the river were multi-resistant. These results allow us to infer the proliferation of β -lactamase genes in Isabela river.

We found common genera between our results and the national health authorities antibiotic resistance surveillance reports; this finding suggests a possible way of transmission between two settings: clinical and environmental. Our results are particularly concerning for local public health authorities as Isabela river's waters are used as a source of drinking water by adjacent communities without proper treatment. Therefore, the data we collected and the conclusions we obtained can be beneficial in the formulation of public health and environmental improvement projects, especially for those living on this river's banks.

## Data Availability Statement

The datasets generated for this study are available on request to the corresponding author.

## Author Contributions

EF, RR, and RB: project conceptualization. VC, RB, CD, and AD: data curation, formal analysis, software, visualization, and original draft writing. OP, LR, and EF: funding acquisition, project administration, and supervision. RB, RR, EF, and VC: methodology. LR, EF, and OP: resources. OP, EF, LR, RR, and RB: review and editing. All authors contributed to the article and approved the submitted version.

## Conflict of Interest

The authors declare that the research was conducted in the absence of any commercial or financial relationships that could be construed as a potential conflict of interest.

## Supplementary Material

Supplementary Figures 1, 2 Dispersion analyses results.

Supplementary Figure 3 Antibiotic resistance of identified clinically relevant strains.

Supplementary Table 1 Physico-chemical parameters of sampled site.

Supplementary Table 2 Complete MALDI-TOF results.

Supplementary Table 3 Complete MIC resistance results.
