# Supplementary material for: Distribution of Beta-Lactamase Producing Gram-Negative Bacterial Isolates in Isabela River of Santo Domingo, Dominican Republic
Source: Front Microbiol. 2021 Jan 13;11:519169. doi: 10.3389/fmicb.2020.519169 (PMC7838461; doi:10.3389/fmicb.2020.519169)
Supplement: Supplementary Figures 1, 2 — Dispersion analyses results. [file Data_Sheet_1.PDF]

Distribution of Beta-Lactamase ProducingGram-Negative Bacterial Isolates in IsabelaRiver of Santo Domingo, Dominican Republic

Supplementary Figures

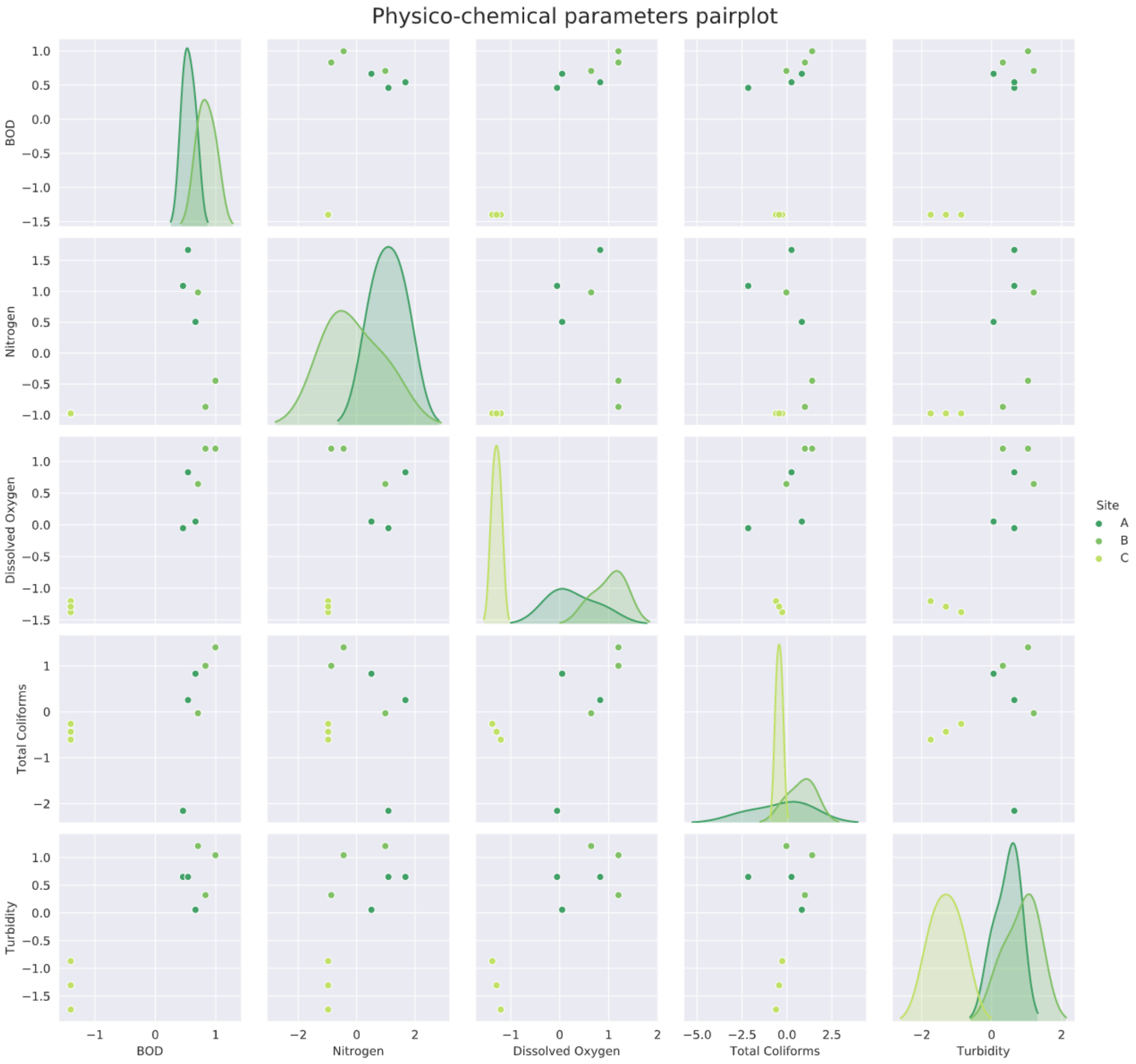

Supplementary Figure 01- Dispersion diagram by pairs of Physicochemical parameters

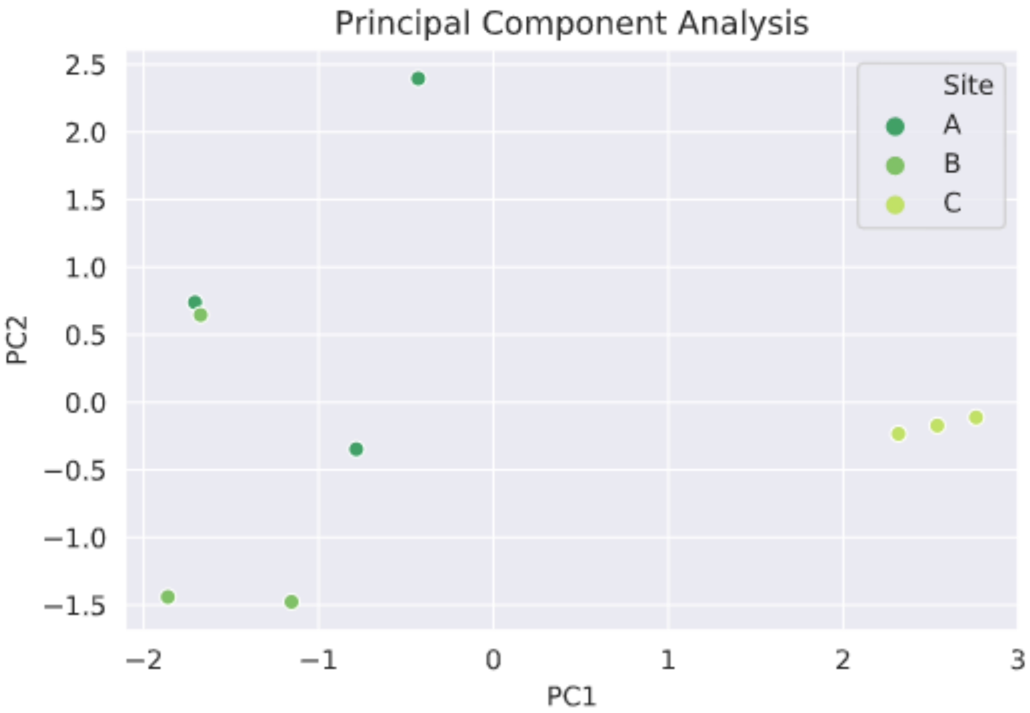

Supplementary Figure 02- Visualization of Clusters by Principal Component Analysis.

The principal component analysis in figure 4 indicates the physicochemical parameters that are most relevant when classifying water. The first component (PC1) explains 73% of the variance using BOD and DBQ as the main physicochemical parameters to discern between regions. The second component (PC2) explains that 21% of the variance in the sample, mostly using the Coliform and Nitrogen parameters to separate the data.

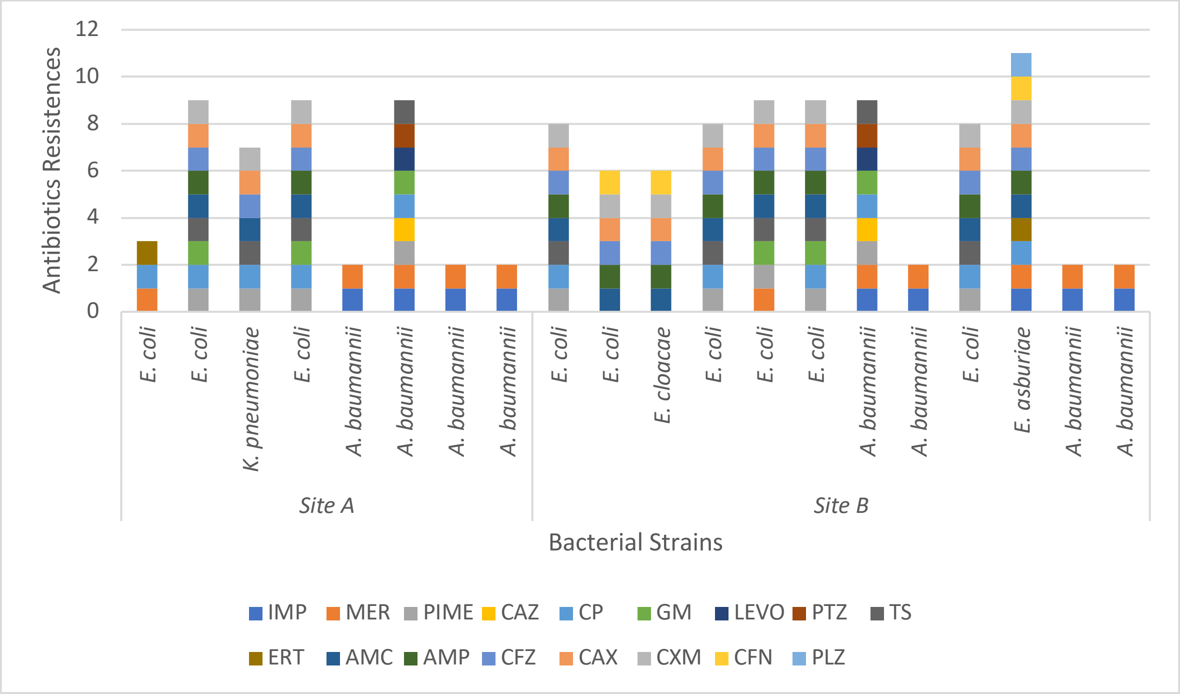

Supplementary Figure 03- Antibiotic resistance of identified clinically relevant strains.
